# Nervous System Response to Neurotrauma: A Narrative Review of Cerebrovascular and Cellular Changes After Neurotrauma

**DOI:** 10.1007/s12031-024-02193-8

**Published:** 2024-02-17

**Authors:** Hossam Tharwat Ali, Idris Sula, Abrar AbuHamdia, Sewar A. Elejla, Ahmed Elrefaey, Hiba Hamdar, Mohamed Elfil

**Affiliations:** 1 Qena Faculty of Medicine, South Valley University, Qena, 83621 Egypt; 2 College of Medicine, Sulaiman Al Rajhi University, Al Bukayriyah, Al Qassim Saudi Arabia; 3 Department of Medical Laboratory Science, Faculty of Medicine and Health Sciences, An-Najah National University, Nablus, Palestine; 4 Alquds University, Jerusalem, Palestine; 5 Ain Shams University, Faculty of Medicine, Cairo, Egypt; 6 Medical Learning Skills Academy, Beirut, Lebanon; 7 Medical University of Plovdiv, Plovdiv, Bulgaria; 8 Department of Neurological Sciences, University of Nebraska Medical Center, Omaha, NE USA

**Keywords:** Neurotrauma, Traumatic brain injury, Cerebrovascular autoregulation, Neuroinflammation, Blood–brain barrier

## Abstract

Neurotrauma is a significant cause of morbidity and mortality worldwide. For instance, traumatic brain injury (TBI) causes more than 30% of all injury-related deaths in the USA annually. The underlying cause and clinical sequela vary among cases. Patients are liable to both acute and chronic changes in the nervous system after such a type of injury. Cerebrovascular disruption has the most common and serious effect in such cases because cerebrovascular autoregulation, which is one of the main determinants of cerebral perfusion pressure, can be effaced in brain injuries even in the absence of evident vascular injury. Disruption of the blood–brain barrier regulatory function may also ensue whether due to direct injury to its structure or metabolic changes. Furthermore, the autonomic nervous system (ANS) can be affected leading to sympathetic hyperactivity in many patients. On a cellular scale, the neuroinflammatory cascade medicated by the glial cells gets triggered in response to TBI. Nevertheless, cellular and molecular reactions involved in cerebrovascular repair are not fully understood yet. Most studies were done on animals with many drawbacks in interpreting results. Therefore, future studies including human subjects are necessarily needed. This review will be of relevance to clinicians and researchers interested in understanding the underlying mechanisms in neurotrauma cases and the development of proper therapies as well as those with a general interest in the neurotrauma field.

## Introduction

Neurotrauma is defined as an injury to the brain or spinal cord that causes significant morbidity and mortality, approximately 3% worldwide (Valle et al. 2022). Physical mechanisms that lead to neurotrauma include impact, impulsive, and static or quasistatic loading as a result of solid object collision, sudden motion, and loading with insignificant effect of speed. They can cause tensile, compressive, and tear damage, which disrupts brain structure and strains its tolerance (Keating and Cullen 2021). It is frequently caused by falls, motor vehicle accidents, and violence, which result in more than 4 million deaths each year (Jullienne et al. 2016; Cash and Theus 2020).

In terms of epidemiology, the incidence of neurotrauma is highest in the high-income countries (GBD 2016 Neurology Collaborators 2019), with males being 40% more affected than females (Gupte et al. 2019). Due to domestic and ambulatory injuries, the prevalence rises after the age of 70. On the other hand, the burden of morbidity and mortality is greatest in the low-income countries, accounting for nearly 90% of the total morbidities and mortalities (Dewan et al. 2018; Raees et al. 2022). Nevertheless, traumatic brain injury (TBI) contributes to more than 30% of all injury-related deaths in the USA and represents more than 75,000 deaths each year in Europe (Jullienne et al. 2016).

Neurotrauma is classified as mild, moderate, or severe from the clinical severity standpoint and as primary or secondary based on the timing of the injury in relation to the insult. Primary injuries are those that occur at the time of the insult and over the ensuing hours and days, whereas secondary injuries develop later after the insult (Smith 2017; Aleman and Prange 2019). The direct results of the mechanical forces of the primary injuries produce deformation and disruption of the brain tissue and function. This includes neuron, axon, glia, and blood vessel damage that initiates a dynamic series of complex alterations at the cellular, inflammatory, mitochondrial, neurochemical, and metabolic levels leading to secondary injuries (McKee and Daneshvar 2015). Secondary injuries cause endoplasmic reticulum (ER) stress, mitochondrial malfunction, and toxic oxidant and nitrogenous species accumulation, which may persist for days or months (Aghili-Mehrizi et al. 2022).

The effects of neurotrauma go beyond the acute manifestations which can include headache, cognitive and behavioral symptoms, and balance and sleep problems, to chronic symptoms that may last for up to 6 months, resulting in complications such as seizures, fatigue, neuroendocrine abnormalities, and hearing and vision problems (Cifu et al. 2015). The main contributor to these long-term problems is the cerebral vascular injury that occurs following neurotrauma and reduces the blood perfusion in the brain at macro- and microcirculation levels (Stephens et al. 2018; Amyot et al. 2019).

Understanding the underlying mechanisms of neurotrauma and neurovascular response to it might help develop effective interventions to improve the clinical outcomes in such patients. In this article, we discuss the nervous system’s response to trauma in light of the existing literature, exposing the various pathways underlying neurotrauma and the emergence of long-term consequences. Furthermore, we point out the gaps in the literature and propose recommendations for future research.

## Nervous System’s Response to Trauma

Traumatic events affecting the central nervous system (CNS) directly and indirectly activate multiple cascades in the CNS ^1^. They trigger immediate cellular and molecular reactions, including inflammatory responses and neuronal circuit disruption ^4^. Neuroinflammation is primarily mediated by glial cells, including astrocytes and microglia. Excessive inflammation in turn may worsen the extent of the injury and impede recovery. Regulation of the neuroinflammatory response to neurotrauma has shown promising effects in the management of a variety of neurological problems ^4^.

On the other hand, neuroplasticity is an adaptive mechanism of the brain to injuries that promotes structural and functional remodeling of the injured neurons ^12^. Structural neuroplasticity involves processes like synaptogenesis, dendritic arborization, and axon sprouting, while functional neuroplasticity encompasses different processes such as cortex remapping, synapse strengthening, and brain network rebuilding. For a long time, it was believed that the CNS had a restricted regenerative capacity when compared to the rest of the body tissues. However, recent studies have demonstrated some regenerative potential, particularly in the peripheral nervous system (PNS) and specific CNS regions such as the hippocampus and olfactory system ^2,5,7^. Understanding the physiological protective mechanisms in the CNS against trauma, such as neuroinflammation and neuroplasticity, could aid in the design of effective interventions and the prevention of long-term complications.

### Cerebrovascular System

#### Cerebrovascular Autoregulation (CA)

The CNS is dependent on steady and maintained cerebral blood flow (CBF) and unlike other organs, it cannot tolerate a shortage of blood supply for a long time. CBF is tightly coupled with neuronal activity so that it is maintained despite changes in systemic blood pressure (Kenney et al. 2016; Charkviani et al. 2019). Cerebrovascular autoregulation (CA) is the primary protective mechanism against any changes in the perfusion pressure. It maintains cerebral perfusion pressure (CPP) within 50–170 mmHg, reducing the effect of fluctuations in the delivery of oxygen and nutrients to neural tissues and protecting them from ischemia and subsequent neurodegenerative abnormalities. CPP is primarily determined by the mean arterial pressure (MAP) and the intracranial pressure (ICP) as CPP = equals the difference between MAP and ICP (CPP = MAP − ICP) (Gleason et al. 2012; Krejza and Arkuszewski 2013; Petkus et al. 2016; Acharya et al. 2022).

**Fig. 1:**
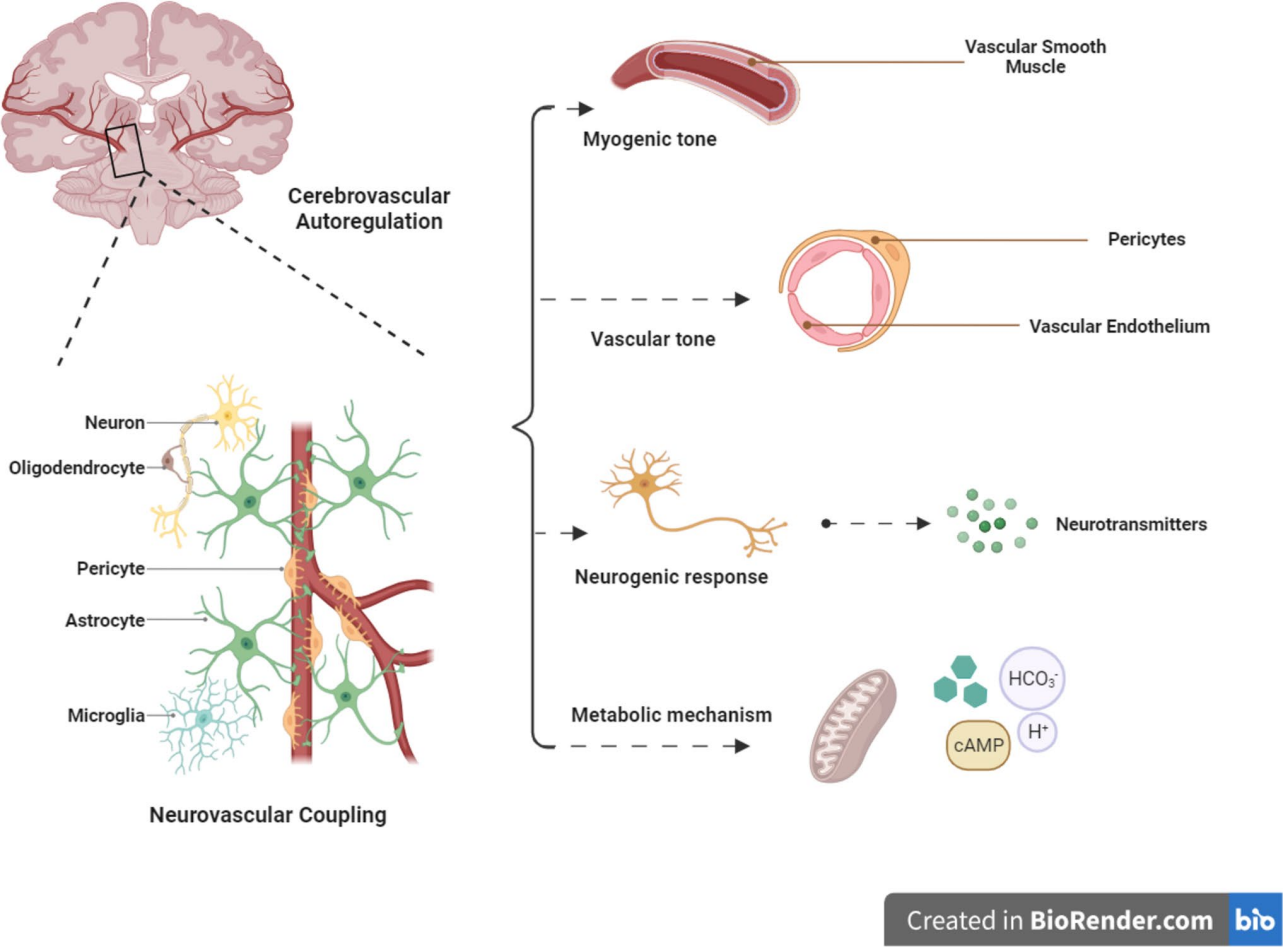
Interacting mechanisms of cerebrovascular autoregulation (CA)

Although the mechanisms of CA are still not well-understood, it is believed to be modulated by four overlapping processes: neurogenic, myogenic, vasogenic, and metabolic processes which require the integration of several components including endothelial cells, junctions, smooth muscle cells, circulating substances, neurons, pericytes, and glial cells (Fig. 1) (Golding 2002; Kenney et al. 2016). For instance, when the blood pressure increases, multiple events start, including increasing pH and the levels of calcium, serotonin, and neuropeptide Y; decreasing osmolarity and the levels of potassium and PaO_2_; and activation of myosin light chain kinase (MLCK) and phospholipase C, leading to vasoconstriction (Tan et al. 2013; Petkus et al. 2016; Vesoulis and Mathur 2017), whereas when blood pressure decreases, PaO_2_, adenosine, osmolarity, potassium, acetylcholine, and nitric oxide increase while pH and calcium ions decrease, leading to vasodilation. It is noteworthy that such mechanisms vary based on the brain region due to regional heterogeneity, with the anterior circulatory system of the brain having denser sympathetic innervation and being controlled primarily by adrenergic sympathetic relays from the superior cervical ganglion via the carotid arteries, whereas the posterior vessels rely on the sympathetic circuit via the vertebrobasilar arteries (Tan et al. 2013; Petkus et al. 2016; Vesoulis and Mathur 2017).

#### Cerebrovascular Changes Post-Trauma

Cerebral vascular dysfunction can precede neuronal damage or occur as a sequel of neuronal dysfunction. The pathology of the cerebrovascular system following TBI can be due to primary injury of the vascular system components or secondary to a pathophysiological cascade that is initiated post-injury (Kenney et al. 2016). Traumatic cerebrovascular injury (TCVI) accounts for around 0.8 to 1.8% of all TBI cases. Although this type of injury necessitates prompt diagnosis and treatment, its diagnosis can be delayed or obscured by an adjacent cerebral injury.

Among the intracranial structures, the cerebral vascular system is particularly vulnerable to injury, even in mild TBI (mTBI), which accounts for up to 90% of TBI cases (Maas et al. 2017; Baker et al. 2022; Drieu et al. 2022). Furthermore, TBI can result in functional disruption and neurodegenerative alterations, which might have an impact on the cerebral vasculature or neurovascular network. Although these disruptions in CBF may be transitory or permanent, they have a substantial impact on cerebral perfusion and post-traumatic complications; they alter CBF even when no anatomical abnormalities are evidently present. However, several investigations that used Doppler ultrasonography found similar anomalies only in the respiratory distress state and not in the relaxed condition (Baker et al. 2022; Das et al. 2022).

TBI impedes the capability of CA which might persist even after the apparent recovery of the patients. For example, TBI might impair the ability of cerebral vessels to dilate which can be very critical when the systemic blood pressure is low which eventually diminishes the CBF (McKee et al. 2014; Charkviani et al. 2019). Along with the diminished CBF, a state of hypermetabolism usually ensues immediately after the biochemical injury to the brain which widens the gap between glucose demand and supply. It is characterized by the rapid release of neurotransmitters; an extracellular glutamate surge has been observed following a brain insult (McGinn and Povlishock 2016). Consequently, this leads to an influx of calcium, efflux of potassium, and sodium–potassium pump hyperactivity to maintain the membrane homeostasis requiring greater supplies of glucose. The increase in intracellular content of calcium ions drives the cellular processes towards apoptotic events through several mechanisms such as the generation of reactive nitrogen and oxygen species, activation of calcium-dependent proteases, and mitochondrial impairment (Saatman et al. 2010; Weber 2012; Cheng et al. 2012). It is believed that such hyperactivity in acute TBI ensues to restore cellular and ionic homeostasis against the progressing neuronal injury. However, this is usually followed by a global decrease in glycolysis that persists until recovery which in humans can take weeks to months (Yoshino et al. 1991; Bergsneider et al. 1997, 2000, 2001; Wu et al. 2004). It is noteworthy that such cascades can occur in diffuse or focal TBI and even in mild TBI as concussions in which no structural abnormalities are detected in conventional imaging (Giza and Hovda 2001; McKee et al. 2014; McGinn and Povlishock 2016). Functional magnetic resonance imaging (MRI) studies have detected persistent altered brain activity in patients with persistent symptoms after mTBI (Chen et al. 2008; Gosselin et al. 2011; McKee et al. 2014).

The decreased density of microvasculature in the cerebral hemispheres of rat models with TBI has been detected along with structural changes to endothelium and pericytes compared to the contralateral normal hemisphere (DeWitt and Prough 2003; Park et al. 2009). In rodents, arterial and venous microthrombi have been detected (Schwarzmaier et al. 2010). It is to be mentioned that smooth muscle cells showed relatively minor or no structural changes or necrosis. Similar changes have been shown in human neuropathological reports of cases with TBI. However, reports of cases dying from fatal TBI are more frequently found except in cases of death after concussion either due to trauma or non-trauma causes (Golding 2002; Kenney et al. 2016).

#### Mechanisms Explaining Alteration in Autoregulation

Several mechanisms can explain the alteration in autoregulation following TBI. Firstly, the inflammatory state resulting from the TBI might lead to an increase in endothelin-1 which antagonizes the N-methyl-D-aspartate (NMDA)–mediated vasodilation which further decreases the CBF (Charkviani et al. 2019). Additionally, endothelin-1 induces an increase in alpha-smooth muscle actin (α SMA) in smooth muscle cells (SMC) and pericytes in the first hours after injury (Jullienne et al. 2016). Moreover, other molecular changes have been observed during the first 48 h after TBI in rat models, such as increased expression of contractile proteins such as calponin associated with enhanced vasoreactivity (Kreipke and Rafols 2009). Furthermore, myogenic mechanisms of autoregulation are often impaired in TBI in which smooth muscles abnormally respond to the pressure changes due to increased protein C kinase and alteration in potential channels of the receptors (Golding et al. 1998; Mathew et al. 1999). Furthermore, nitric oxide (NO) plays a significant role in vascular tone along with the myogenic mechanisms and is considered the primary endogenous vasodilator in the brain (Kenney et al. 2016). NO in low concentrations exhibits favorable effects in the form of vasodilation. However, in high concentrations, it mediates free radical–mediated pro-inflammatory effects. Moreover, NO synthase (NOS) shows a bimodal fluctuation post-injury which further contributes to the altered autoregulation and CBF (Jullienne et al. 2016).

Even though the myogenic regulation and the role of vascular SMCs and endothelial cells in maintaining cerebral perfusion are involved in the vascular tone, the control of perfusion in intraparenchymal arterioles is also influenced by “intrinsic” innervation coming from the surrounding brain neuropil, including interneurons and astrocytes (Jullienne et al. 2016). A few studies have detected a decrease in perivascular innervation due to a decrease in nerve fibers in perivascular space which impedes the neurogenic regulation of the vascular tone (Duff et al. 1986; Sercombe et al. 2002; Ueda et al. 2006). In such cases, there was a decreased response to vasoactive substances (Wei et al. 1980; Armstead 1997; Fujita et al. 2012).

On the chronic scale, chronic traumatic encephalopathy (CTE) is characterized by focal perivascular deposition of p-tau in the neocortex that can spread to the adjacent cortex, TDP-43 accumulation, neuronal and axonal loss, and cerebral atrophy. Axonal injury and degeneration play a critical role in the initiation of p-tau pathology (McKee et al. 2014, 2015; McKee and Daneshvar 2015). Microvasculopathy in the form of hyaline changes has been detected since the first cases were reported (McKee et al. 2014; Kenney et al. 2016). Further studies are required to thoroughly investigate the underlying mechanisms and consequences on the chronic level (McKee and Daneshvar 2015; Baker et al. 2022).

#### The Blood–Brain Barrier (BBB) Changes Post-Trauma

The BBB is formed by the tight connections between endothelial cells and regulates the transport of blood-borne factors through the paracrine function of astrocytes and endothelial cells. One of the most serious consequences of TBI is the disruption of the BBB which varies according to patients’ age and the type and severity of the injury (Jullienne et al. 2016; Charkviani et al. 2019). Disruption of the BBB was previously thought to be a short-term event that occurs within hours of injury and normalizes within the duration of a week. However, further research has demonstrated that the pathological changes of the BBB following TBI might persist up to years after the injury (Jullienne et al. 2016; Cash and Theus 2020). Studies on stroke rat models showed opened BBB due to endothelial changes and increased contractile protein expression up to one- and 2 months post-injury respectively (Strbian et al. 2008; Pop et al. 2013). It is worth pointing out that long-term neurodegeneration after trauma might be in part due to disturbance in the BBB’s function and/or structure. Early restoration of the BBB’s integrity helps prevent long-term sequelae of trauma such as neurodegenerative changes (Pop et al. 2013; Cash and Theus 2020).

**Fig. 2:**
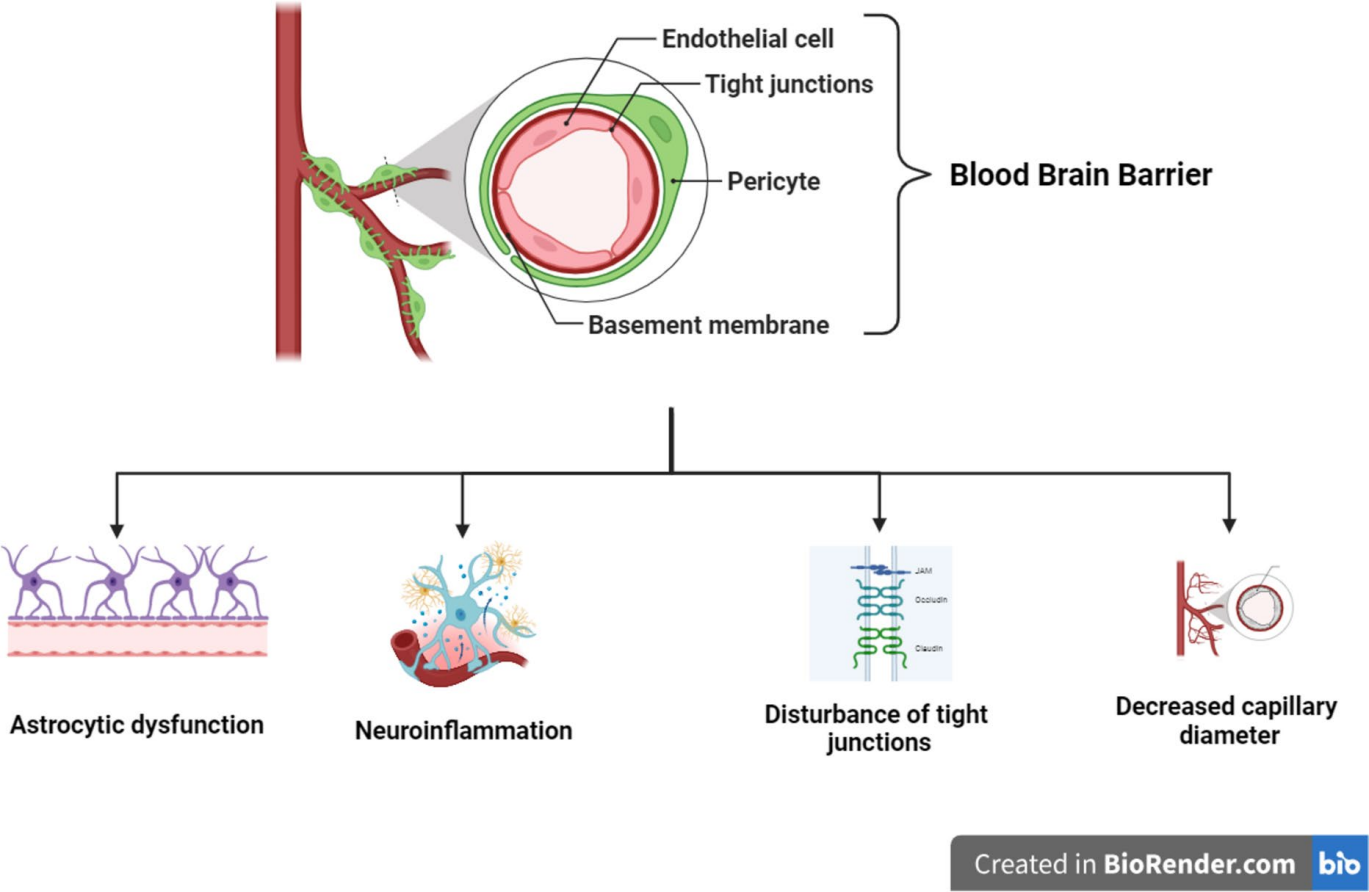
Changes in blood–brain barrier (BBB) following traumatic brain injuries (TBI)

The immediate pathological change of the BBB following TBI is the disruption of its tight junctions causing an increase in paracellular permeability (Fig. 2). Extravasation of the serum protein, fibrinogen, and immunoglobulin G, both markers for BBB disruption, was observed in the brains of human patients who died in the acute phase following TBI, as well as in those who survived at least a year. An influx of immune cells such as neutrophils also occurs which further exacerbates the inflammatory response (Hay et al. 2015; Cash and Theus 2020). Additionally, disruption of endothelial ion transporters ensues. Increased secretion of sodium ions into the neurons drives in more water resulting in edema. Furthermore, injury causes oxidative stress and increased production of inflammatory cytokines and proteins which along with the increased permeability leads to vasogenic edema. Accumulation of fluid in perivascular spaces also occurs which alters the CBF and increases the ICP (Charkviani et al. 2019; Cash and Theus 2020).

Furthermore, injury to the endothelial cells and astrocytes is involved in impairing the cellular metabolism and ion transporters which can lead to cytotoxic edema. Moreover, in patients with persistent late inflammatory state after TBI (> 14 days), endothelial transcytosis significantly contributes to increased BBB permeability. It is to be taken into consideration that the changes in BBB may similarly occur in microvessels of the brain due to the systemic inflammatory process (Jullienne et al. 2016; Charkviani et al. 2019; Cash and Theus 2020).

#### Mechanisms of Cerebrovascular Repair

The endogenous mechanisms of cerebrovascular repair in TBI are not fully understood. Nevertheless, hypoxia is believed to induce angiogenesis and other hypoxia-inducible factors. Deposition of extracellular matrix proteins and thickening of basement membrane and vascular wall usually ensue within hours after TBI (Golding 2002; Kenney et al. 2016). A recent study on mouse models of mTBI showed that angiogenesis started within a few days through infiltrating myeloid cells (Russo et al. 2018). Pharmacological therapies that promote angiogenesis have been shown to improve neuronal outcomes even if administered late in rat models (Kenney et al. 2016). Note that a subsequent TBI on day 1 after the initial TBI impaired the recovery process while on day 4, it did not (Russo et al. 2018; Baker et al. 2022).

A study on rats revealed vascular repair with increased vascular endothelial growth factor-A (VEGF-A) expression (Park et al. 2009). In humans, screening mTBI patients with computed tomography (CT) abnormalities revealed increased circulating VEGF-A levels (Edwards et al. 2020). Moreover, athletes with a history of mTBI were shown to have increased VEGF-A levels compared to control groups (Major et al. 2020). Of clinical relevance to this mechanism, regular subsymptom exercise was shown to promote the healing process, improve symptoms, and reduce recovery time in acute and subacute settings of mTBI (Leddy et al. 2019; Baker et al. 2022). On a cellular level, VEGF induces changes in vascular smooth muscle cells that lead to alteration in arterial contractility and an increase in the muscle cell’s synthetic or secretive function in terms of releasing extracellular matrix proteins (Jullienne et al. 2016).

### Autonomic Nervous System (ANS) Involvement in TBI

ANS is a part of the PNS that is composed of sympathetic and parasympathetic divisions that regulate all organ functions and maintain homeostasis, including thermoregulation, respiration rate, and pupillary response via autonomic and somatic reflexes (McCorry 2007; Takahashi et al. 2015). Severe TBI results in a specific pattern of autonomic dysfunction that affects cortical and subcortical control pathways. TBI stimulates the ANS to release catecholamines and inflammatory signals, which exaggerate the inflammatory response and increase morbidity and mortality. Thus, patients with autonomic dysfunction as a result of TBI must be evaluated, monitored, and controlled as quickly as possible (Takahashi et al. 2015; Esterov and Greenwald 2017).

The most prevalent autonomic impairment following TBI is paroxysmal sympathetic hyperactivity. Exaggerated vital signs, such as tachycardia, tachypnea, hyperthermia, and hypertension, are typical in patients with that condition (Perkes et al. 2010). The specific mechanism behind these occurrences has yet to be established. A study using an excitatory-inhibitory model observed lesions in the mesencephalic area, which were thought to obstruct inhibitory pathways from the brain, activating peripheral sympathetic nervous systems (Fernandez-Ortega et al. 2012). On the other hand, other studies claimed that TBI overstimulates the excitatory center in the brainstem by directly damaging it (Perkes et al. 2010; Fernandez-Ortega et al. 2012). Further investigation is underway to determine the most likely root of autonomic dysfunctions.

Autonomic dysfunction has also been seen in cases of subarachnoid hemorrhage, and it is thought to be caused by a spike in inflammatory cytokines after injury. Brain injuries activate the sympathetic nervous system, causing the autonomic nervous system to secrete pro-inflammatory cytokines, resulting in neuroinflammation and neurodegeneration (Fernandez-Ortega et al. 2012; Purkayastha et al. 2019). Furthermore, these injuries raise ICP, causing aneurysms to develop in the walls of brain vessels and aneurysmal rupture. As a result, these injuries disturb the functioning of the hypothalamus, which receives information from the upper cortical centers and sends it to the brainstem and spinal cord, altering the cortical centers and disrupting the autonomic nervous system. This disruption further stimulates the ANS, resulting in primary injury to the brain and remote organs. Such a cascade can occur even in patients with minor trauma (Naredi et al. 2000; Purkayastha et al. 2019; Lee and Jang 2021).

Although various mechanisms have been proposed to explain the disruption of the ANS following TBI, more research on the mechanism and characteristics of autonomic dysfunction in TBI, particularly mild TBI, is needed to better understand the link between autonomic dysfunction and other post-TBI sequelae and to improve diagnostic and therapeutic approaches.

### Neuroinflammation: Cellular and Molecular Changes

Although traditionally known as “immunologic privilege,” parenchymal inflammation “neuroinflammation” can be triggered even in mild cases of TBI where cerebrovascular disturbance may not exist, and conventional imaging cannot detect it (McGinn and Povlishock 2016; Drieu et al. 2022). A study on mouse models with mTBI showed that a single mTBI event can induce parenchymal inflammatory response in the form of astrogliosis and microgliosis up to 3 weeks after the injury which was consistent with findings from human biopsies (Engel et al. 2000; Willis et al. 2020; Witcher et al. 2021). Several studies revealed that microglia can show an inflammatory state weeks or months after the acute effects of the injury have disappeared. Such hyperactivity of microglia can predispose the patient to chronic degenerative changes and morbidity besides the increased vulnerability to subsequent insults (McGinn and Povlishock 2016). Along with the local inflammatory response, recruitment and infiltration of immune cells into the CNS also occur due to the release of the pro-inflammatory cytokines. The concomitant endothelial changes and BBB structural or functional disruptions allow early invasion of neutrophils and monocytes which mediates the acute phase of inflammation (Clark et al. 1994; Holmin et al. 1998).

#### Pericytes

Pericytes are part of the neurovascular unit and are considered multifunctional cells that are embedded in the capillary walls all over the body including the brain (Brown et al. 2019). They are also referred to as “the hidden regulators” of the vascular system through many vascular functions including cerebral blood flow control, blood–brain barrier maintenance, and angiogenesis development. In addition, it facilitates the neuroinflammatory process and has stem-like properties (Yamazaki and Mukouyama 2018). Due to this important role, it has increasingly been recognized as a contributor to the development of many cerebral disorders including TBI especially in the early stages (Dalkara et al. 2011; Wu et al. 2022). Its response to injury includes capillary constriction, recruitment of immune cells, and regulation of the immune response. Activated pericytes contribute to tissue repair by secreting various growth factors, such as VEGF and basic fibroblast growth factor (bFGF), which promote angiogenesis and neurogenesis. Moreover, activated pericytes can modulate the activity of nearby immune cells. In addition, it proliferates and migrates to the scar’s formation in the damaged area and resupplies it with nutrients. Therefore, pericytes have been targeted nowadays for potential therapeutic approaches (Dalkara et al. 2011; Yamazaki and Mukouyama 2018; Cheng et al. 2018). However, excessive activation can lead to pathologic changes in the blood vessels, such as capillary constriction and BBB disruption (Dalkara et al. 2011; Yamazaki and Mukouyama 2018). These changes might worsen ischemia, increase brain edema, and exacerbate neuroinflammation, all of which are detrimental to neural recovery. Thus, pericytes are believed to be involved in the development of chronic conditions such as vascular cognitive impairment (Nikolakopoulou et al. 2017).

#### Glial Cells

Glial cells, also known as neuroglia, support and protect neurons in the CNS. They provide structural support, insulation, and chemical equilibrium. Astrocytes, oligodendrocytes, and microglial cells are examples of glial cells (Mietto et al. 2015).

##### Astrocytes

Astrocytes are the most abundant glial cells and their functions include providing structural and metabolic support to neurons by controlling the composition of extracellular fluid, the BBB, ion concentrations, synaptic activity, and the control of CBF (Baaklini et al. 2019). Astrocytes play a major role in the event of neurovascular coupling by releasing vasoactive substances, such as prostaglandins and epoxyeicosatrienoic acids (EETs) to relax the smooth muscle cells surrounding blood vessels, leading to vasodilation (Niu et al. 2019; Lyu et al. 2021). Moreover, they regulate extracellular potassium levels by taking up excess potassium ions, as well as taking up excess neurotransmitters like glutamate and converting them to maintain the energy balance required for neuronal activity (Lyu et al. 2021). Astrocytes also play a role in the long-term regulation of CBF. Astrocytes release different molecules, such as VEGF and NO, which help in influencing the tone of blood vessels and help maintain CBF. They can regulate the permeability of the BBB and indirectly influence CBF by controlling the entry of molecules that affect vascular function (Stephens et al. 2018).

Reactive astrocytes are specialized astrocytic cells within the CNS that undergo distinct molecular and morphological changes following CNS injury (Lyu et al. 2021). They exhibit multifaceted roles including formation of gliotic scar and tissue remodeling and serving as a structural barrier by isolating injured tissue from the healthy neural milieu (Amyot et al. 2019). Reactive astrocytes can dispense factors that support synaptic plasticity and repair, by facilitating the re-establishment of functional synaptic connections following an injury. By alternating the extracellular matrix constituents, reactive astrocytes direct cell migration patterns and axonal growth during tissue remodeling processes (Stephens et al. 2018; Lyu et al. 2021). Furthermore, studies on rat models showed a significant reduction in the astrocyte population after the injury (Hill-Felberg et al. 1999; Zhao et al. 2003). Some studies showed compensating restoration of the astrocyte count after 1 month (Hill-Felberg et al. 1999).

##### Oligodendrocytes 

Oligodendrocytes are myelin producers. Myelin sheaths improve the effectiveness of brain communication by facilitating the transfer of electrical signals between neurons. Furthermore, they promote and maintain axon integrity (Niu et al. 2019; Morel et al. 2020). Myelin sheaths play a crucial role in response to neurotrauma, which varies depending on the type and severity as well and location (central or peripheral) of the trauma. Myelin sheaths provide protection and insulation for axons. In cases of minor neurotrauma, myelin sheaths act as shields for axons from direct damage, thus reducing the risk of axonal injury (Mei et al. 2016; Baaklini et al. 2019). However, neurotrauma might disrupt or damage myelin sheaths resulting in the loss of myelin integrity and impairing the conduction of nerve impulses in affected axons (Patrikios et al. 2006). This might result in a temporary or permanent loss of function in the injured area. Severe neurotrauma cases, such as TBI or spinal cord injury (SCI), can lead to widespread demyelination along affected axons. This can result in significant functional deficits and neurological symptoms. Oligodendrocytes initiate remyelination in response to injury to the CNS. Successful remyelination might result in partial or even full recovery of function. However, severe and extensive demyelination might result in long-term or permanent loss of function (Franklin and Kotter 2008; Mei et al. 2016; Morel et al. 2020).

##### Microglial Cells

Microglial cells are one of the CNS immune cells, and they serve as the initial line of defense. They respond to traumas and inflammations, as well as remove damaged cells, thereby regulating the brain environment (De Biase and Bonci 2019; Estell 2021). Understanding the roles of glial cells is critical for understanding the complex interactions inside the brain and their role in a variety of neurological conditions. Microglial cells get activated in response to neurotrauma and release pro-inflammatory molecules, such as cytokines and chemokines to clear debris and pathogens from the injured area. However, prolonged inflammation might lead to secondary tissue damage. Activated microglia can phagocytose cellular debris, dead neurons, and foreign substances at the site of injury (De Biase and Bonci 2019; Lyu et al. 2021). This helps in removing damaged tissue and clearing toxins (Kettenmann et al. 2013). Microglial cells can switch from pro-inflammatory (M1) to anti-inflammatory (M2) states depending on the microenvironment and the stage of injury (Crain et al. 2013). The balance between these states is crucial for the resolution of inflammation and tissue healing. Following neurotrauma, microglial cells become activated as part of the innate immune response. They migrate to the site of injury and change their morphology to survey the damaged area (Kettenmann et al. 2013; Lyu et al. 2021). Microglial cells play a role in the formation of glial scars in response to injury. The glial scars play a protective and inhibitory role in nerve regeneration (Li et al. 2021). Ongoing research aims to better understand microglial responses and develop strategies to modulate their activities for improved outcomes in cases of neurotrauma.

#### Growth and Neurotrophic Factors

The cellular responses to a TBI event are distinguished by various features, the most important of which are the contributions of growth and neurotrophic factors. Growth factors are specific sorts of proteins that are responsible for cell development and repair. They are released after a TBI event to promote the formation of new blood vessels, a process known as angiogenesis, to restore blood flow to affected areas. On the other hand, neurotrophic factors promote neuronal development. After a TBI event, brain cells release neurotrophic factors to promote neuronal survival, the regeneration of new neurons known as neurogenesis, and neural connections (Huebner and Strittmatter 2009; Morel et al. 2020). Growth and neurotrophic factors are critical in the repair and recovery of vascular and neuronal brain tissue. Overall, growth factors promote angiogenesis and hence improve blood supply to injured areas, whereas neurotrophic factors increase neuroplasticity and neurogenesis (Mietto et al. 2015; Wofford et al. 2019; De Biase and Bonci 2019).

Growth factors and neurotrophic factors play crucial roles in the response to neurotrauma, such as TBI or SCI. After neurotrauma, growth factors and neurotrophic factors promote the survival of the injured neurons by the activation of signaling pathways that inhibit apoptosis and prevent further neuronal loss in the injured site (Kahn and De Vellis 1995). In cases of nerve injury or SCI, growth factors and neurotrophic factors stimulate and facilitate the regrowth of axons and the re-establishment of neural pathways. Neurotrophic factors, particularly brain-derived neurotrophic factor (BDNF), are critical for synaptic plasticity. VEGF plays a key role in angiogenesis to provide oxygen and nutrients to injured tissues and facilitate the repair and recovery process. Growth factors and neurotrophic factors have significant therapeutic potential in neurotrauma; however, their use in clinical applications is still an active area of research (Kahn and De Vellis 1995; Nikolakopoulou et al. 2017; Wofford et al. 2019).

## Secondary Mechanisms of Injuries in Neurotrauma

Despite the extensive research and substantial improvement in the management of neurotrauma, secondary injuries in the form of chemical and molecular responses to primary injuries are considered inevitable (Aghili-Mehrizi et al. 2022). Secondary injuries, persisting for days to months, can have more serious and life-long implications than the primary events (Centers for Disease Control and Prevention (CDC) 2013; Osier et al. 2015). Up till now, there is no Food and Drug Administration (FDA)–approved drug to prevent or control the secondary mechanisms after nervous system injuries (Ismail et al. 2020). ER stress, mitochondrial dysfunction, and oxidative stress are the major mechanisms for secondary injuries (Aghili-Mehrizi et al. 2022).

### ER Stress

The ER has essential functions such as lipid biosynthesis, calcium storage, and protein folding and modification (Iurlaro and Muñoz-Pinedo 2016). Neurotrauma, causing oxidative stress, neuroinflammation, and metabolic disturbance, can lead to the accumulation of unfolded proteins and thus ER stress and dysfunction (Larner et al. 2006; Zhang and Kaufman 2008; Oakes and Papa 2015; Wu et al. 2016). To restore protein homeostasis, the unfolded protein response (UPR) pathway is initiated. UPR works to reduce the unfolded protein through alterations in protein expression. In the acute stage, UPR can restore ER and cellular homeostasis. However, high or prolonged stress states can lead to UPR-associated apoptosis (Cao and Kaufman 2012; Lucke-Wold et al. 2014; Smith and Wilkinson 2017; Read and Schröder 2021). Furthermore, in such conditions, the ubiquitin–proteasome system (UPS), which is responsible for degrading proteins targeted for destruction, can be affected leading to aggregated ER dysfunction and disease process (Bence et al. 2001; Imai et al. 2001; Sokka et al. 2007). Notably, many studies detected elevated levels of UPR markers following brain and spinal injuries (Larner et al. 2004; Sokka et al. 2007; Lucke-Wold et al. 2014, 2016; Wu et al. 2016). Agents targeting the UPR pathway showed promising results in animals where they have been shown to increase cerebrovascular perfusion while decreasing ER stress–mediated apoptosis, neuroinflammation, and so neural deficits (Vaccaro et al. 2013; Donnelly et al. 2013; Logsdon et al. 2014; Cho et al. 2015; Rubovitch et al. 2015; Dash et al. 2015; Lucke-Wold et al. 2017; Hood et al. 2018; Tan et al. 2018; Ruiz et al. 2020).

### Mitochondrial Dysfunction

In neurotrauma, reduced blood and oxygen supply lead to inhibition of aerobic metabolism. This makes mitochondria upregulate the anaerobic metabolism through the production of lactate to compensate for the reduction in energy production (Ghosh et al. 2017; Greco et al. 2020). Mitochondrial dysfunction can exist with any apparent ischemic changes due to the excitotoxic effects of accumulated Ca^+2^ which acts through stimulation of NMDA receptors (Carvajal et al. 2016; Luo et al. 2019). Consequently, voltage-gated Ca^+2^ receptors are opened leading to an influx of calcium and activation of calcium-dependent proteases and production of reactive oxygen species (Carvajal et al. 2016; Mattson 2017; Luo et al. 2019; Kumar Sahel et al. 2019). Furthermore, disruption in calcium homeostasis causes outer membrane permeabilization (MOMP), the formation of mitochondrial permeability transition pores (mPTP), and eventually cell death (Giorgi et al. 2012; Millet et al. 2016). Recent studies focusing on extrasynaptic NMDA receptors, which promote cytoplasmic calcium and mitochondrial stress, have shown that memantine downregulates such receptors and protects against mitochondrial and neuronal damage in the settings of TBI in rodent models (Bernardi 1996; Wang et al. 2018). Another promising option is that it targets the inhibition of mPTP through reproducing the effects of cyclosporin A, a well-established molecule in preventing apoptosis (Halestrap et al. 1997; Scorrano et al. 1997; Springer et al. 2018).

### Oxidative Stress

Free radicals are unstable chemicals that are formed through covalent bond disruption and can react with other free radicals or molecules (Ljubisavljevic 2016). Under physiologic conditions, various enzymes such as superoxide dismutase (SOD), catalase, and glutathione peroxidase exert antioxidant actions preventing serious effects of free radicals (da Silva Meirelles et al. 2017). Secondary injury in neurotrauma through various cascades, including those discussed above in this review, leads to excessive free radical formation, further exacerbating injury. The excessive formation of free radicals exceeds the capacity of antioxidant mechanisms, creating a vicious cycle of free radical formation. Eventually, free radicals interact with lipids, proteins, nucleic acids, and carbohydrates causing irreversible oxidative damage (Bains and Hall 2012; da Silva Meirelles et al. 2017; Ismail et al. 2020). Having high lipid concentrations and abundant oxidative metabolism, the CNS is specifically sensitive to oxidative damage. Due to apoptosis, changes in the CNS include neuroinflammation, mitochondrial dysfunction, edema, and BBB disruption allowing the invasion of immune cells which exaggerates the injuries (Ljubisavljevic 2016; Wang et al. 2018). Scavenging agents of free radicals such as edaravone, which is approved for ALS treatment, have been shown to reduce apoptotic activity in rodent models of TBI (Wang et al. 2011; Ismail et al. 2020). Other tested drugs on rodent models include apocynin, an NADPH oxidase inhibitor, and TBHQ, an NRF2 activator that could salvage gray and white matter after TBI (Ünal et al. 2020). Additionally, mitoquinone, an antioxidant that targets the mitochondrial electron transport chain, has recently been investigated for TBI although its efficacy for neurodegenerative diseases has been widely studied (Zhou et al. 2018; Ismail et al. 2020).

## Role of the Glymphatic System in Neurotrauma

The glymphatic system is a recently discovered brain-wide network that facilitates waste clearance from and nutrient delivery to the brain. In contrast to the lymphatic system, the glymphatic system is located only in the brain (Wang et al. 2017; Tian et al. 2022). Although the CNS is considered by many to be an immunologically privileged organ, the interaction between the brain tissue and the immune system was unclear with the presence of BBB. However, the late discovery of the glymphatic system gave a clearer picture (Lindblad et al. 2020; Tian et al. 2022).

TBIs can disrupt the glymphatic flow and lead to waste accumulation resulting in inflammation and secondary injuries (Lindblad et al. 2020; Piantino et al. 2021). Astrocytes might be damaged in a TBI event and result in an impaired ability of glymphatic to clear up wastes from the brain. The accumulation of different proteins including tau and beta-amyloid, which are associated with neurodegenerative diseases, can be exacerbated by glymphatic dysfunction following a trauma event (Lindblad et al. 2020). In rodents experiencing a mild TBI, the loss of perivascular aquaporin-4 polarization in astrocytes led the glymphatic dysfunction and impaired waste clearance from the brain tissues, which potentially contributes to waste accumulation resulting in post-traumatic neurodegeneration (Piantino et al. 2021). Puhakka et al. reported brain-enriched proteins in the deep cervical lymph nodes following experimental TBI, suggesting their clearance through the glymphatic system (Puhakka et al. 2023).

Wang et al. reported that multiple microinfarcts caused by the intraarterial injection of cholesterol crystals resulted in a wide disruption of CSF inflow along the glymphatic route (Wang et al. 2017). Even though the glymphatic dysfunction was temporary, CSF tracers kept accumulating within the surrounding brain tissue. This illustrates a more subtle disruption of glymphatic system function (Wang et al. 2017). Moreover, glymphatic system dysfunction due to the impact of microinfarcts was more aggravated among old rodents (1 year) as compared to younger rodents (2–3 months). This suggests that the glymphatic system’s functions are more vulnerable in the aging brain as compared to younger brains experiencing microinfarcts (Wang et al. 2017; Benveniste et al. 2019). Similarly, a recent study revealed post-TBI edema owing to the suppression of glymphatic flow. Adrenergic receptor inhibition, using a combination of prazosin, atipamezole, and propranolol, almost eliminated the cerebral edema which improved neurological and behavioral outcomes (Hussain et al. 2023).

There are several ways to study the glymphatic flow and functions in post-TBI brains with MRI and positron emission tomography (PET) being the most used (Piantino et al. 2021). Moreover, the investigation of potential biomarkers, particularly specific proteins, or metabolites, can help in identifying glymphatic dysfunction in trauma patients. A better understanding of the glymphatic system can open the way for the development of targeted therapies that can enhance glymphatic flow which holds immense potential for improving brain health and developing novel therapeutic approaches for various neurological disorders including neurotrauma.

## Clinical Implications and Challenges

Neurotrauma has a striking variability in clinical presentation. The severity and type of symptoms can range from mild post-concussive symptoms to focal neurologic deficits (Dennis et al. 2022; Yue and Deng 2023). This variety makes diagnosis difficult because the same underlying vascular event can manifest differently in different people. Given the heterogeneity in patient characteristics and pathoanatomical subtypes, determining the extent of acute injury and long-term prognosis remains difficult (Yue et al. 2023). For example, patients with cerebrovascular neurotrauma can have vastly different trajectories, making accurate prediction difficult. Thus, due to clinical heterogeneity, research in cerebrovascular neurotrauma faces significant challenges. When dealing with a condition that has numerous different manifestations, designing effective clinical trials becomes difficult (Frisvold et al. 2023). Understanding this diversity is critical for the development of targeted therapies and the improvement of outcomes. Management may include addressing respiratory issues, providing nutritional support, and using specific interventions such as hypertonic saline or mannitol as needed (National Academies of Sciences, Engineering, and Medicine; Health and Medicine Division; Board on Health Care Services; Board on Health Sciences Policy; Committee on Accelerating Progress in Traumatic Brain Injury Research and Care 2022). It is critical to tailor treatment to each patient’s specific needs. Subsequently, due to its inherent heterogeneity, neurotrauma poses clinical challenges. Understanding and addressing this diversity are critical for improving diagnosis, prognosis, and treatment in this highly specialized field.

### Clinical Challenges with Neurotrauma in Terms of Population Differences

It is becoming increasingly clear that the impact and management of neurotrauma vary significantly across different populations, with factors such as gender, age, and the presence of pre-existing medical diseases influencing the clinical outcomes of TBI (Gupte et al. 2019). Significant gender differences in neurotrauma have been discovered through research. According to studies, women often have poorer outcomes after a TBI than men (Blaya et al. 2022). They may have a higher rate of psychosocial challenges and internalized disorders, especially if there is a history of childhood brain injury (McKinlay et al. 2002). Understanding these gender disparities is critical for designing effective interventions and support systems.

Furthermore, age has a significant impact on neurotrauma outcomes. Mild closed-head injuries in children and adolescents can cause behavioral and cognitive issues, emphasizing the importance of early intervention (McKinlay et al. 2002). Older adults, on the other hand, are more vulnerable to falls and age-related brain injuries, necessitating specialized care and rehabilitation programs. Recognizing age-related challenges in neurotrauma care is critical for improving outcomes (Blaya et al. 2022). When dealing with neurotrauma, patients who have pre-existing medical conditions face additional challenges. TBI diagnosis and management may be complicated by conditions such as Alzheimer’s disease. Understanding the interaction between neurotrauma and pre-existing medical comorbidities is critical for providing comprehensive care and reducing complications (Smith et al. 2021).

Finally, addressing the issues of neurotrauma necessitates a nuanced understanding of how gender, age, and medical diseases intersect with this complex condition. To provide equitable care for all individuals affected by neurotrauma, tailored interventions and support systems must be developed.

## Limitations of Available Studies and Future Directions

Numerous studies on TBI have been conducted, ranging from in vitro studies using culture and animal models to human studies. Animal models were used to discover new concepts, linkages between phenotype and genotype, long-term outcomes, and complications and to pave the way for clinical trials. However, when it comes to translating the findings of these studies into human language, especially the neurobehavioral aspects, several issues arise, particularly anatomical differences. The development and structure of the brain, particularly the cortical layers, vary across species. Studies often use lissencephalic species due to their lower operational complexity, costs, and ethical issues compared to gyrencephalic species, despite differences in brain structure, surface, and size (Sun and Hevner 2014; Jullienne et al. 2016; Cash and Theus 2020).

Various animal models, including weight drop (WD), fluid percussion (FP), and controlled cortical impact (CCI), have been used, each with its own set of limitations. WD and FP injuries lack standardization, impacting cognitive deficits and reproducibility. They do not depict all clinical signs of TBI, disrupt the brainstem, cause apnea, and can falsify TBI. The controlled cortical impact (CCI) model improves injury control by allowing precise injury depth, timing, and velocity. However, it requires head stabilization and anesthesia, unlike human TBI. Anesthetics like isoflurane and ketamine protect the brain and improve outcomes in TBI models. The closed-head impact model of engineered rotational acceleration (CHIMERA) is a novel method that uses isoflurane anesthesia to study head movement during impact, assessing brain tissue damage. However, CHIMERA, an animal model, lacks an accurate representation of human TBI due to its use of isoflurane, which may improve TBI injuries, necessitating further research for new models that represent human TBI accurately. Understanding the limitations of each model allows us to better interpret brain damage (Okonkwo et al. 2017; Petersen et al. 2021).

Culture models study molecular, cellular, and subcellular mechanisms. However, static mechanical systems like transection, compression, and barotrauma lack quantification of force, strain, and strain rate, while dynamic mechanical systems like acceleration/deceleration and cell stretch have limitations as they cannot measure tissue deformation. Moreover, cell-stretch models are expensive and require highly qualified expertise. The use of uniaxial, biaxial, and 3-D injury systems for TBI is complex and requires specialized expertise, making them unsuitable for mild injuries. Other cell culture systems, like extraparenchymal tissue, immortalized cell lines, and primary cultures, face challenges in harvesting and culturing and are costly (Morrison et al. 2011; Adelson et al. 2011). Animal studies have identified factors linked to CNS regeneration post-TBI, including myelin-associated inhibitors, chondroitin sulfate proteoglycans, repulsive guidance molecules, and semaphoring 3A. Further research is needed to evaluate these factors in humans and determine their long-term effects on neurons and their signaling.

Lastly, human studies were done to confirm the findings of cultural and animal model findings, yet they still had a few limitations. Observational studies have recall bias and information bias due to a lack of standard protocols. Experimental studies lack long-term outcomes, proper control, and confounders and are unrepresentative due to small sample sizes, lack of coverage, and poor protocols (Adelson et al. 2011; Okonkwo et al. 2017; Chesnut et al. 2018; Horton et al. 2018; Tunthanathip et al. 2019; Gao et al. 2020). For instance, recent research has given insight into some therapies that showed promise in treating increased ICP, such as decompressive craniectomy (DC), including lateral DC, bifrontal DC, and primary DC. However, they are still not well-understood and require further investigation (Tunthanathip et al. 2019; Hawryluk et al. 2020). Furthermore, studies reveal that TBI is caused by oxidative stress, which affects brain cells, particularly astrocytes, and the inflammatory response, which is a natural defense mechanism against injuries. Immune cells are key modulators of the inflammatory response, but their actions and interactions are not fully understood. Researchers should exercise caution when interpreting results from animal and culture models in humans, as immune cells differ in various species and situations (Wofford et al. 2019; Jaganjac et al. 2022).

Cerebrovascular injuries following TBI, including microbleeds and CBF dysfunction, contribute to poor outcomes and neurodegeneration (Dewan et al. 2018). However, they are still obscure and affected by age and sex; future studies must count on these interferences to improve personalized treatments (Huebner and Strittmatter 2009; Jullienne et al. 2016; Baker et al. 2022). Precise diagnosis and monitoring are crucial for managing TBI, but most pediatric neuroimaging studies focus on early childhood stages, with limited representation of infants and toddlers. Future research is needed to understand the underlying mechanisms of TBI and its potential long-term effects and to develop more effective diagnostic tools (Lindsey et al. 2019).

## Conclusions

Neurotrauma encompasses a complex and multifaceted clinical landscape with heterogeneity in presentation from one patient to another. TBI is a worldwide public health problem with associated significant morbidity and mortality. Despite extensive research, effective interventions for neurotrauma remain elusive. Understanding the vascular, cellular, and molecular sequalae of neurotrauma is crucial to designing effective studies and tailoring efficient treatments. Thus, the underlying pathophysiological and repair mechanisms are yet to be fully described. Moreover, there is a need to translate animal studies into human studies to improve better management of TBI.

## Data Availability

No datasets were generated or analyzed during the current study.

## Abbreviations

TBI: Traumatic brain injury
ER: Endoplasmic reticulum
CNS: Central nervous system
CBF: Cerebral blood flow
CA: Cerebral autoregulation
MLCK: Myosin light chain kinase
MAP: Mean arterial pressure
ICP: Intracranial pressure
CPP: Cerebral perfusion pressure
CVR: Cerebral vascular resistance
TCVI: Traumatic cerebral vascular injury
NMDA: N-Methyl-D-aspartate
SMA: Smooth muscle actin
SMC: Smooth muscle cells
NO: Nitric oxide
NOS: Nitric oxide synthase
BBB: Blood-brain barrier
VEGF-A: Vascular endothelial growth factor-A
CT: Computed tomography
ANS: Autonomic nervous system
bFGF: Basic fibroblast growth factor
EETs: Epoxyeicosatrienoic acids
SCI: Spinal cord injury
BDNF: Brain-derived neurotrophic factor
FDA: Food and Drug Administration
UPR: Unfolded protein response
UPS: Ubiquitin-proteasome system
MOMP: Mitochondria outer membrane permeability
mPTP: Mitochondrial permeability transition pores
SOD: Superoxide dismutase
PET: Positron emission tomography
WD: Weight drop
FP: Fluid percussion
CCI: Controlled cortical impact
CHIMERA: Closed-head impact model of engineered rotational acceleration
